# Electromyographic activity of the vastus medialis and gastrocnemius implicates a slow stretch-shortening cycle during rowing in the field

**DOI:** 10.1038/s41598-020-66124-4

**Published:** 2020-06-11

**Authors:** Held Steffen, Siebert Tobias, Donath Lars

**Affiliations:** 1 Department of Intervention Research in Exercise Training, German Sport University Cologne, Cologne, Germany; 2 Department of Sport and Motion Science, University of Stuttgart, Stuttgart, Germany

**Keywords:** Physiology, Musculoskeletal system

## Abstract

The consideration of the temporal and electromyographic (EMG) characteristics of stretch-shortening cycles (SSC) are crucial for the conceptualization of discipline-specific testing and training. Since leg muscles are first stretched (eccentric) and then contracted (concentric) during rowing, it can be assumed that the entire muscle tendon complex performs a SSC. Thus, it should be elucidated whether the rowing cycle can be attributed to either a slow or fast SSC. Therefore, EMG of the *vastus medialis* and *gastrocnemius* were captured (n = 10, 22.8 ± 3.1 years, 190 ± 6 cm, 82.1 ± 9.8 kg) during (*single scull*) rowing and subsequently compared to typical slow (countermovement jump, CMJ) and fast (drop jump, DJ) SSCs. The elapsed time between the EMG onset and the start of the eccentric phase was monitored. The pre-activation phase (PRE, before the start of the eccentric phase) and the reflex-induced activation phase (RIA 30–120 ms after the start of the eccentric phase) have been classified. Notable muscular activity was observed during DJ before the start of the eccentric phase (PRE) as well as during RIA. In contrast, neither CMJ nor rowing revealed any EMG-activity in these two phases. Interestingly, CMJ and race-specific rowing showed an EMG-onset during the eccentric phase. We conclude that rowing is more attributable to a slow SSC and implies that fast SSC does not reflect discipline specific muscle action and could hamper rowing-performance-enhancement.

## Introduction

The sequence of stretching and contraction of a muscle tendon unit (MTU) is described as a stretch-shortening cycle (SSC)^1^. The resulting muscular force, work and power during a SSC achieve up to 50% higher values, compared to an isolated concentric contraction^2–4^. Increased muscular efficiency and decreased metabolic costs have been discussed to account for these findings^5,6^. The increased muscle performance during a SSC is however still not completely understood^7^. The performance enhancement during a SSC can be attributed to (a) the storage and release of elastic-energy^8,9^, (b) stretch-induced contractility enhancement^7,10^ and (c) reflex activity and time-to-peak force^11,12^. In addition, a differentiation between slow (longer than 250 ms, such as countermovement jump, CMJ) and fast SSC (shorter than 250 ms, such as drop jumps, DJ) needs to be considered within discipline-specific movement analyses^13,14^. DJ are characterized by the three distinctive characteristics of the fast SSC: pre-activation (before the eccentric phase), reflex activity and an SSC duration below 250 ms^1,13,15,16^. In contrast to this, CMJ are characterized by SSC duration above 250 ms and a lack of pre-activation and reflex-activity, which are the characteristics of slow SSC^1,13,15,16^.

In this regard, the rowing-cycle can be classified to a propulsive phase (drive) and a gliding-phase (slide). During one rowing-cycle, the legs are undergoing a flexion (slide), followed by an extension pattern (drive)^17^. The assumption that the entire muscle tendon complex performs a SSC during this flexion-extension cycle is confirmed by eccentric muscle activity during the late gliding-phase before the start of a new rowing stroke^18–22^. In rowing, jumps are trained over the entire year and thus constitute a central component of the testing and training schedules of rowers^23–28^. In this context, fast (such as DJ) and slow SSC movements (such as CMJ) are frequently applied^23,29^. However, the potential SSC in rowing can only correspond to one of these two SSC types. In addition, each of these SSC types represent independent dimensions (in terms of movement amplitudes and velocities), what is also reflected in a training-methodological differentiation of slow and fast SSC: Training adaptations in the fast SSC are not necessarily transferable to performance increases in the slow SSC (and vice versa)^1,13,16,30^. Accordingly, the combined training of fast and slow SSC movements results in an unnecessarily high physiological workload for the rowing athletes. Consequently, this substantial physiological workload can be reduced by classifying the SSC type in rowing.

Against this background, the present study was conceptualized and conducted in order to elucidate, whether the potential SSC in rowing comprise either a fast or a slow SSC (of the entire MTU): In the course of this, the surface electromyographic muscle activity (sEMG) during in-field rowing (*single scull*) should be compared to slow (CMJ) and fast (DJ) SSC. Due to the fact that fast SSC in contrast to slow SSC are characterized by pre-activation and reflex activity^1,15,16^, sEMG data comparison of DJ, CMJ, and rowing enable the separation between fast and slow SSC in rowing. The underlying design was based on the assumption that sport-specific muscle-actions are required during training^1,16,30^. Since the longer SSC duration in rowing^31^ (compared to DJ) is an indication of slow SCC, the hypothesis of this study was that rowing is characterized by a lack of pre-activity and reflex activity and therefore attributable to a slow SSC. It should be noted that it is not the duration of the SSC but the characteristic features (reflex activity and pre-activation) that are the crucial criteria for differentiating between slow and fast SSC^1,13,15,16^. To the best of our knowledge, this is the first study that employed those methods under field conditions. The results of the study might gain notable impact on the conceptualization of rowing specific testing and training, using appropriate exercises that adequately reflect electro-mechanical properties of SSC in rowing.

## Methods

### Participants

Ten experienced male rowers of the national squad (22.8 ± 3.1 years, 190 ± 6 cm, 82.1 ± 9.8 kg, 3 male lightweight rowers, 7 male heavyweight rowers, at least 5 years of training experience) were enrolled in the present cross-sectional study. Each of the participants already won international medals at Rowing World Championships (u19, u23 or elite) and 8 out of 10 participants were world champions in their respective competition classes. No health impairments were reported at the time of the investigation. The participants were familiar with jump (DJ and CMJ) testing and training. Declarations of consent were obtained from all participants. All methods were carried out in accordance with relevant guidelines and regulations. The study protocol has been approved by the local ethical committee (Ethics Committee - German Sport University Cologne, 172/2018) and fulfilled the international ethical standards^32^.

### Study design

**Figure 1:**
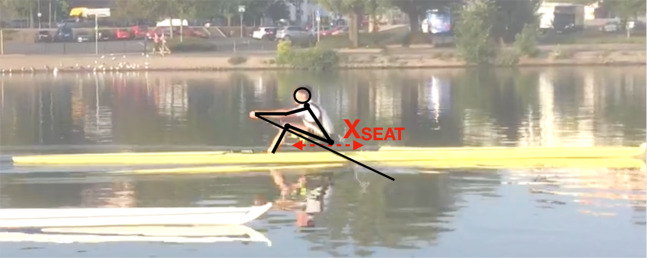
Schematic representation of rowing. (X_seat_ ≙ seat-position; written informed consent was obtained from the individual for the publication of this image.) During a rowing-stroke, the leg-, trunk- and arm-muscles work in sequence. The muscles work temporarily simultaneously during the transitions between the leg-, trunk- and arm-working phases. The beginning of the seat-movement (during the slide phase) indicates the start of the eccentric phase.

After a standardized 10-minute warm-up program (10 min running at low intensity/heart rate, which corresponds to a blood lactate concentration <2 mmol/L and about three practice jumping trials), DJ, CMJ, low intensity (LiR) and high intensity rowing (HiR) were performed in a randomized order. DJ (32 cm drop) and CMJ are characterized by high test-retest reliabilities^33^. For each jump type, five scoring attempts were allowed, after three habituation trials. Between all scoring jumps (DJ and CMJ) a break of 2 min was maintained. The field rowing measurements (Fig. 1) were carried out in the *single scull*, in order to exclude interactions between the athletes. For data acquisition, two 500 m trials with LiR and HiR were performed. The HiR trials were carried out with an intensity, corresponding to the 2000 m rowing competition. There was a break of 5 min between each trial. The participants were instructed to complete all rowing trials with a constant stroke-rate (LiR: 20 strokes per minute, HiR: 36 strokes per minute). The stroke-rate was shown to the athletes by a display (Stroke Coach, Nielsen-Kellerman, USA) as live-feedback. In order to minimize external influences (e.g. shipping traffic and water stream), the measurement took place in a harbour basin. Since athletes had to reach the required target rowing-speed (and stroke-rate), 10 consecutive stroke cycles within the last 60 seconds of each 500 m trial were considered.

### Data sampling and analyses

The time-interval from the beginning of the eccentric phase (T_ECC_) to the end of the concentric phase (T_CON_) was referred as SSC-Time (T_SSC_). T_SSC_ of DJ, CMJ, LiR and HiR were calculated, based on the recorded ground contact times during DJ (contact-mat, Refitronic, Schmitten, Germany), the center-of-mass (CoM) movement during CMJ (pull-wire sensor, Refittronic, Schmitten, Germany, accuracy ± 0.5%) and the seat movement (pull-wire sensor, Refittronic, Schmitten, Germany) during (LiR and HiR) rowing. In addition, T_CON_ and T_ECC_ were determined during CMJ, LiR and HiR. The jump heights were calculated using the flight time (T_Flight_) method^34^. The reliability of the flight time method in combination with the used contact-mat was high^35^.

In addition to these kinematic data, the following sEMG analysis were conducted: The two leg muscles *m. vastus medialis* (EMG_VM_) and *m. gastrocnemius medialis* (EMG_GM_) were examined using sEMG during in-field rowing (LiR and HiR) and compared with DJ and CMJ. During muscle shortening, the *m. vastus medialis* stretches the knee in order to contribute to leg extension. As an ankle joint plantar flexor, the *m. gastrocnemius* further contributes to leg extension. Moreover, due to the fixation of the feet on the stretcher (foot-plate of the boat) (*Lombard’s paradox*)^36^, the bi-articular *m. gastrocnemius medialis* seems to work as a leg extensor in the knee joint angle range of 150 to 180°^37^. Pairs of Ag/AgCl circular bipolar, pre-gelled surface electrodes (Paediatric Red Dot, 3 M, Minnesota, USA) were attached to the skin with a fixed 20 mm inter-electrode distance. Skin preparation and electrode placement followed the SENIAM recommendations^38^. A reference electrode for differential signal recording was fixed on the fibula head. All electrodes remained fixed at the same place during the entire measuring process. sEMG signals were amplified using miniature amplifiers (BioVision, Wehrheim, Germany), converted by a 24-bit AD-board (DaQ 700, National Instruments, Austin Texas, USA) and stored continuously on a computer using a commercially available software package (Dasylab, National Instruments, Austin Texas, USA). The sampling rate was 1000 Hz. As recommended in previous research^39^, the EMG signal was bandpass filtered (10–500 Hz). In addition, artefacts and noises of the EMG signal were visually examined. The sEMG data were triggered as follows: The ground contact-mat (Refftronic, Schmitten, Germany) was used to detect the first ground contact during DJ, which triggers the start of eccentric phase during DJ. The CoM movement during CMJ was recorded, using a pull-wire sensor (Refitronic, Schmitten, Germany) to trigger the sEMG signal. In this context, the beginning of downward movement of the CoM was used to define the start of the eccentric phase at CMJ. A pull-wire sensor (Refitronic, Schmitten, Germany, accuracy ± 0.5%) was used to detect the beginning of the seat-movement (during the slide-phase), which triggers the start of the eccentric phase during (LiR and HiR) rowing. This experimental setup subsequently compared and evaluated the sEMG data regarding the reflex activity, whereby the differentiation of arbitrary and reflex-related sEMG-activity was based on the work of Gollhofer and co-workers^16,40,41^: Overall, a distinction was made between the pre-activation phase (PRE, 150 ms before the start of the eccentric phase till the start of the eccentric phase), the reflex-induced activation phase (RIA 30–120 ms after the start of the eccentric phase) and the late-EMG- response (LER, 120 ms after the start of the eccentric phase till the end of the concentric phase)^16,40–42^. EMG_VM_ and EMG_GM_ indicate the onset of sEMG-activity relative to the beginning of the eccentric phase. As an additional reference system (EMG_VM-TP_ and EMG_GM-TP_), the turning-point of the CoM was used in rowing (front-turn) and CMJ (lowest position), i.e. the point in time when the eccentric phase has finished, and the concentric phase starts. The sEMG onsets were determined when the amplitude of the sEMG signal exceeds three standard deviations over a baseline amplitude for more than 20 ms^22^. Furthermore, it was checked whether the sEMG-activities fall below the respective onset-limits (corresponding to the previously defined limit-value of onset-determination) within the concentric phases. In addition, the magnitude of sEMG activity was determined by the root mean square (RMS) of PRE- (RMS_GM-PRE_ and RMS_VM-PRE_), RIA- (RMS_GM-RIA_ and RMS_VM-RIA_) and LER-phase (RMS_GM-LER_ and RMS_VM-LER_). For each muscle RMS were described relative to the maximum values (%-max) of the respective subjects, measured over the whole test period^22^.

### Statistics

All data are presented as group mean ± standard deviation. The collected T_SSC_ and sEMG onsets were examined with the *Kolmogorov-Smirnov*-*Test* for normal distribution and the *Levene*-*Test* for variance homogeneity. Separate repeated measurement analysis of variance (rANOVA) were applied to the different test conditions DJ, CMJ, EXA and WSA, using EMG_GM_, EMG_VM_, EMG_GM-TP_, EMG_VM-TP_, RMS_VM-PRE_, RMS_VM-RIA_, RMS_VM-LER_, RMS_GM-PRE_, RMS_GM-RIA_, RMS_GM-LER_, T_CON_, T_ECC_, and T_SSC_ as within subject variable, respectively. *Bonferroni* Post-hoc-Tests were subsequently applied for pairwise comparison. rANOVA effect sizes were given as η_p_^2^ with values ≥0.01, ≥0.06, ≥0.14 indicating small, moderate, or large effects, respectively^43^. Standardized mean group differences as a measure of pairwise effect size estimation were also calculated (SMD, trivial: d < |0.2 | , small: |0.2 | ≤ d < |0.5 | , moderate: |0.5 | ≤d < |0.8 | , large d ≥ |0.8 | )^43^. Statistical analyses were performed using a statistic software package (IBM SPSS Statistics, Version 25.0, Armonk, NY, United States). Moreover, a *p*-value below 0.05 was considered as statistically significant.

## Results

### Kinematic parameters

Compared to the T_SSC_ of DJ, the SSC-duration was about 3 times, 5 times, and 7 times of that value for CMJ, LiR, and HiR, respectively (Table 1). Based on the T_FLIGHT_, jump heights of 30 ± 5 cm for CMJ and 25 ± 5 cm for DJ were calculated. The rANOVA revealed significant differences (*p* < 0.001, η_p_^2^ > 0.138; Table 1) between DJ, CMJ, LiR, and HiR, which applied to the within subject variables T_CON_, T_ECC_, and T_SSC_. The pairwise post-hoc-tests showed significant (*p* < 0.05) increases and large effect sizes (SMD > | 0.8 | ) of T_SSC_ from DJ to CMJ to HiR to LiR. Also, T_CON_ and T_ECC_ increased significantly (*p* < 0.05, SMD > | 0.8 | ) from CMJ to HiR to LiR.

**Table 1:** Overview (means ± standard deviation) of SSC-Duration (T_SSC_), Flight-Times (T_FLIGHT_), duration of the eccentric phase (T_ECC_), duration of concentric phase (T_CON_), sEMG-activity-onset relative to the start of eccentric phase (EMG_GM_ and EMG_VM_), sEMG-onset relative to the center-of-mass turning-point (EMG_GM-TP_ and EMG_VM-TP_), magnitude (RMS) of sEMG (RMS_VM-PRE_, RMS_VM-RIA_, RMS_VM-LER_, RMS_GM-PRE_, RMS_GM-RIA_ and RMS_GM-LER_) at drop jumps (DJ), countermovement jumps (CMJ), low intensity rowing (LiR) and high intensity rowing (HiR).

	DJ	CMJ	LiR	HiR	*p*	*η*_p_^2^
T_SSC_ (ms)	257 ± 78	731 ± 217	1869 ± 162	1203 ± 135	***	+++
T_FLIGHT_ (ms)	457 ± 44	497 ± 40	—	—	***	+++
T_ECC_ (ms)	—	435 ± 79	987 ± 149	555 ± 68	***	+++
T_CON_ (ms)	—	296 ± 166	882 ± 43	648 ± 110	***	+++
EMG_GM_ (ms)	−138 ± 22	369 ± 77	1068 ± 69	473 ± 127	***	+++
EMG_VM_ (ms)	−108 ± 33	251 ± 130	1044 ± 102	426 ± 119	***	+++
EMG_GM-TP_ (ms)	—	−66 ± 104	81 ± 133	−82 ± 118	**	+++
EMG_VM-TP_ (ms)	—	−184 ± 179	57 ± 63	−129 ± 54	***	+++
RMS_GM-PRE_ (%)	28.2 ± 12.5	2.3 ± 0.9	2.4 ± 1.1	2.5 ± 1.0	**	+++
RMS_GM-RIA_ (%)	68.0 ± 32.3	2.7 ± 0.9	2.9 ± 0.7	2.3 ± 1.0	***	+++
RMS_GM-LER_ (%)	51.2 ± 18.6	34.8 ± 15.9	30.8 ± 15.5	28.6 ± 14.5	0.070	+++
RMS_VM-PRE_ (%)	14.6 ± 9.1	2.9 ± 0.7	2.7 ± 0.8	2.5 ± 0.5	**	+++
RMS_VM-RIA_ (%)	73.8 ± 20.1	2.6 ± 1.0	2.8 ± 0.7	2.9 ± 0.8	***	+++
RMS_VM-LER_ (%)	67.5 ± 21.0	43.8 ± 27.9	29.4 ± 10.4	36.7 ± 17.9	*	+++

The significances (***p < 0.001, **p < 0.01, *p < 0.05) and effect sizes (+++: η_p_^2^ ≥ 0.138, ++: η_p_^2^ ≥ 0.059, +: η_p_^2^ ≥ 0.01) of the repeated measurement ANOVA are given.

### sEMG onset-related parameters

Between the diagnosed sEMG-onsets and the end of the concentric phase, the sEMG-activity of DJ, CMJ, LiR, and HiR did not fall below the established limit for the sEMG-onset-determination (for 20 consecutive ms). The rANOVA revealed significant differences (*p* < 0.01, η_p_^2^ > 0.138; Table 1) between DJ, CMJ, LiR, and HiR, which applied to EMG_GM_, EMG_VM_, EMG_GM-TP_, and EMG_VM-TP_. The pairwise post-hoc-tests showed no significant (*p* > 0.05) differences but large effect sizes (SMD > | 0.8 | ) of EMG_VM_ and EMG_GM_ between CMJ and HiR. Apart from this, significant (*p* < 0.05) increases and large effect sizes (SMD > | 0.8 | ) of EMG_VM_ and EMG_GM_, from DJ to CMJ to LiR were observable. In addition, EMG_VM-TP_ (*p* > 0.05) and EMG_GM-TP_ showed no significant (*p* > 0.05) difference and only trivial effect sizes (|0.14 | > SMD < | 0.47 | ) between CMJ and HiR. In contrast, significant (*p* < 0.05) higher values and large effect sizes (SMD > | 0.8 | ) of EMG_VM-TP_ and EMG_GM-TP_ were observed during LiR, compared to CMJ and HiR.

**Figure 2:**
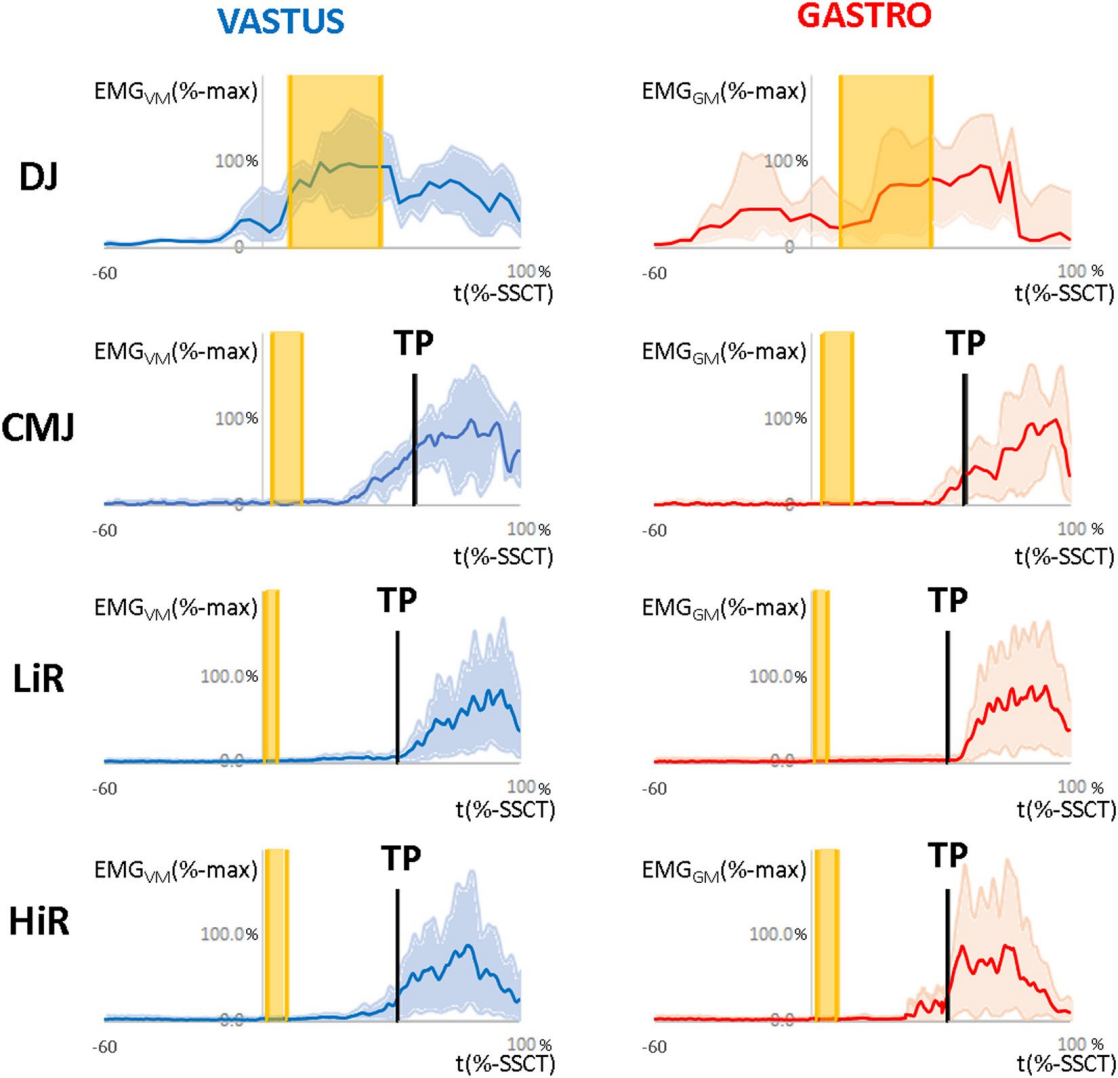
Presentation of sEMG data (mean ± 95% confidence interval) of the entire sample during drop jumps (DJ), countermovement jumps (CMJ), low intensity rowing (LiR), and high intensity rowing (HiR). sEMG activity of the *m. gastrocnemius medialis* (EMG_GM_) was displayed in red. EMG activity of the *m. vastus medialis* (EMG_VM_) was displayed in blue. The rectified sEMG signal was shown as root mean square (RMS, 30 ms). The RMS amplitude was normalized to the respective maximum (%-max). Accordingly, the eccentric phase of each movement (DJ, CMJ, LiR and HiR) begins at 0% and the concentric phase ends at 100%. In addition, center-of-mass turning points (TP) were marked for CMJ, LiR and HiR. The reflex induced activation phase (RIA) was marked in yellow.

Based on visual inspection (Fig. 2), the sEMG-signals (EMG_GM_ and EMG_VM_) showed a clear muscular activity before the first ground-contact (PRE) in the reflex-induced phase (RIA) and in the late EMG response phase (LER) during DJ. On the other hand, neither CMJ nor (LiR and HiR) rowing showed an sEMG-activity (EMG_GM_ and EMG_VM_) in the PRE or RIA phase. Only the LER could be confirmed in all investigated muscle actions (DJ, CMJ, LiR, and HiR). In addition, the onset (in relation to the CoM-turning point) of the EMG_VM-TP_- and EMG_GM-TP_-activity during LiR appeared significantly after the start of the concentric phase. In contrast, the sEMG activity (EMG_VM-TP_ and EMG_GM-TP_) in CMJ and HiR begun significantly before the CoM-turning-point.

### RMS-related parameters

Regarding the RMS data, the rANOVA revealed significant differences (*p* < 0.05, η_p_^2^ > 0.138; Table 1) between DJ, CMJ, LiR, and HiR, which applied to the within subject variables RMS_VM-PRE_, RMS_VM-RIA_, RMS_VM-LER_, RMS_GM-PRE_, and RMS_GM-RIA_. The pairwise post-hoc-tests showed no significant (*p* > 0.05) differences and only trivial effect sizes (SMD < | 0.2 | ) of RMS_VM-PRE_, RMS_VM-RIA_, RMS_GM-PRE_, and RMS_GM-RIA_ between CMJ, LiR and HiR. In contrast, DJ showed significant (*p* < 0.05) higher RMS_VM-PRE_, RMS_VM-RIA_, RMS_GM-PRE_ and RMS_GM-RIA_ values and large effect sizes (SMD > | 0.8 | ), compared to CMJ, LiR and HiR. While RMS_VM-LER_ of DJ revealed also significant (*p* < 0.05) higher values and large effect sizes (SMD > | 0.8 | ) then LiR and HiR, no significant (*p* > 0.05) differences but large effect sizes (SMD > | 0.8 | ) between DJ and CMJ were observed for RMS_VM-LER_. In addition, RMS_VM-LER_ showed no significant (*p* > 0.05) differences between LiR and HiR. In the context of RMS_GM-LER_, the rANOVA revealed no significant (*p* > 0.05) difference but large effect sizes (η_p_^2^ > 0.138) between DJ, CMJ, LiR, and HiR. Overall, the RMS data showed high muscular activities during DJ in all three phases (PRE, RIA and LER). In contrast, CMJ and rowing (LiR and HiR) showed negligible muscular activity during the PRE and RIA.

## Discussion

To the best of our knowledge, this was the first study that addressed whether rowing comprise either a fast or a slow SSC (of the entire MTU) during rowing under field conditions employing surface electromyography. We observed that the sEMG-signals of the *m. gastrocnemius medialis* and *m. vastus medialis* during DJ showed notable muscular activity during pre-activation-phase, reflex-induced-activation-phase and late-EMG-response-phase. In contrast, neither the CMJ nor the rowing pattern itself (high- and low-intensity) showed an sEMG activity (*m. gastrocnemius medialis* and *m. vastus medialis*) during the pre-activation-phase or reflex-induced-activation-phase. Only the late EMG response (*m. gastrocnemius medialis* and *m. vastus medialis*) could be confirmed in all investigated muscle actions (DJ, CMJ, LiR, and HiR). In summary, slow SSC (CMJ) and race-specific high intensity rowing (HiR) showed a sEMG-onset during the eccentric phase. In contrast, the sEMG-onset during fast SSC (DJ) appears before the start of the eccentric phase (before first ground contact). Moreover, the sEMG-onset (*m. vastus medialis*, EMG_VM-TP_ and *m. gastrocnemius medialis*, EMG_GM-TP_) showed no significant difference between CMJ and HiR rowing (in relation to the start of the eccentric phase). In addition, the measured SSC times (Table 1) indicated that potential SSC in field single scull rowing comprises only slow SSC. Consequently, the potential SSC (of entire MTU) in rowing is most-likely attributable to a slow SSC, which is characterized by a lack of pre-activation and reflex-activity^1,15,16^.

In general, the relationship between the neuromuscular response and the sEMG-onset depends on the corresponding mechanisms of the respective muscle activity: The characteristic sEMG-onset (of DJ) before the first ground contact^44^ is described as pre-activity. This pre-activity provides optimal preparation of the working muscles and is regarded as a part of a central movement control program^45^, which is triggered on supraspinal level^46^. The amplitude of this muscular pre-activity is proportional to the drop height and is timed to the expected time of ground contact^47^. Based on Dietz^48^ it can be concluded that the pre-activity of drop jumps depends on the expectation of the moment of ground contact. In addition, the duration of pre-activity in DJ correlates with the muscle stiffness during the early phase of ground contact and the short latency stretch reflex of the vastus lateralis^49^. Accordingly, the pre-activity appears to generate the necessary basic activation on which the effectiveness of the subsequent reflex responses depends. During DJ, reflex activities can be detected in the sEMG signal (about 40–45 ms after the first ground contact)^50^, which are additionally added to the arbitrary neuronal activation. Zuur and colleagues^51^ changed the time of ground contact during DJ by changing the height of the floor surface, while the participants were airborned and unaware of this procedure. The resulting reflex activity was exactly time-locked to the instant of ground contact: Lowered landing areas leaded to longer latencies and lifted landing areas leaded to shorter latencies. These observations confirmed the stretch-induced reflex activity during DJ. In (fast) SSC movements, these reflex activities are considered to be an important factor in the ability to transfer energy from the pre-activated and eccentrically stretched muscle–tendon complex to the concentric phase^45,52^. The first arbitrary sEMG activities can occur about 120 ms after the first ground contact^53^. In general, only in case of unpredictable muscle actions (e.g. twist one’s ankle) a time-based EMG examination can be clearly separated between voluntary and reflex-induced muscle activity^54,55^. The pre-activation during DJ can be initiated quiet deliberately (arbitrarily). However, since no sEMG muscle activity was observed during the PRE and RIA phases (Fig. 2), meaningful pre-activation and reflex activity during CMJ and rowing can be ruled out.

Numerous studies showed^56,57^ that SSC performance can be increased mainly by reactive force capabilities, induced by adequate training (e.g., plyometrics). In the context of plyometric training (SSC related training) in rowing, contradictory research results exist: While one intervention study (n = 18, 4 weeks) revealed rowing-specific performance improvements through plyometric training^28^, another intervention study (9 weeks, n = 24) observed no rowing-specific performance improvements^27^. These contradictory results might be explained by methodological issues: One Study^28^ was volume matched but not workload matched and the performance level of the participants (in both studies)^27,28^ was very low (500 m time trial mean power was about 45% lower than the performance of the participants in this current study). In addition, both studies used variations of slow (CMJ) and fast (DJ) SSC movements. Unfortunately, training adaptations in the fast SSC were not directly transferable to the slow SSC^1,13,16,30^. Together with the current results (the potential SSC in rowing is attributable to a slow SSC), this might implicate that any forms of muscle action in the fast SSC do not reflect discipline specific muscle actions and could hamper rowing performance enhancement during training and competitions. In order to adapt the frequently used plyometric training in rowing^24–28^ to the current results, the fast SSC movements (DJ, depth jumps, box to box jumps) should be replaced by slow SSC exercises (variations of CMJ, Box Jumps, hurdle jumps or similar). Since the EMG activity prior to the onset of a new rowing stroke was only observable during high intensity rowing and the training intensity distribution in rowing is dominated by low intensity rowing^58–60^, short rowing sprint intervals with a high stroke rate and intensity should be performed more frequently^26^. In addition, the increase of maximum strength capability should be the main goal of strength training in rowing, since the slow SSC is predominantly determined by the dynamically realized maximum force^61^. However, it should be noted that the maximum strength requirements in rowing are subject to an optimal trend^62^. Accordingly, the shift in training paradigm in rowing from endurance-oriented strength training to less stressful hypertrophy-oriented strength training forms^63^ (as e.g. realized in the performance centre in Dortmund [Germany]) can be further corroborated by the results of this work.

One limitation of the current study is that eccentric and concentric phases were not quantified during the DJ. The CoM movement during the DJ was recorded with a pull-wire sensor. However, this data could not be evaluated, because the cable (due to the fast movement) started to oscillate or was ruptured. Accordingly, future research should use alternative measurement methods. In case of dynamic contractions, the innervation zone position changes its position during time^64^. Together with the different joint angles of the separate tasks (DJ, CMJ, LiR and HiR), this influenced the sEMG amplitude results. In addition, the pennation of the medial gastrocnemius could result in a locally muscular activity^65^. Therefore, the electrode location could affect the onset estimation and the detected sEMG activation of the gastrocnemius muscle^66^. These effects were reduced by keeping the electrode position constant during all measurement trials. Regarding the onset detection, the determination of the eccentric phase is not precise, because the beginning of the eccentric phase of gastrocnemius and vastus medialis was not identical during DJ (depending on the landing technique). However, due to large differences (about 0.5 s or greater) between DJ, CMJ and rowing, small differences (a few ms) had low effects on the sEMG onset results. Nevertheless, small differences at the start of the eccentric phase may have affected the RMS data as the PRE and RIA phases may have overlapped. Since the sEMG onsets for CMJ and rowing were clearly detected after (approx. 130–1070 ms) the PRE or RIA phase, only minor distortions of the RMS data were to be expected. Based on the current investigation it cannot be precisely determined whether the muscle fasciae actually perform an SSC during rowing. However, due to the eccentric muscle activity during the late gliding-phase prior to the start of a new rowing stroke^18–22^, SSC mechanisms for rowing can be assumed. Future research should investigate the verification of the SSC in rowing. In this context, sEMG, goniometer and ultrasound measurements of the fascicle’s operating length and velocity as well as the activation of a leg extensor muscle during rowing are currently in preparation.

In summary, slow SSC (CMJ) and race-specific high intensity rowing (HiR) showed a sEMG-onset during the eccentric phase. In contrast, the sEMG-onset during fast SSC (DJ) appeared before the start of the eccentric phase (before the first ground contact). In conclusion, our investigation showed that the potential SSC in rowing is attributable to a slow SSC. These findings might implicate that any forms of muscle action in the fast SSC domain do not reflect discipline specific muscle actions and could hamper rowing performance enhancement during training and competitions.

## Data Availability

All data generated or analysed during this study are included in this published article. Apart from this the datasets generated during and/or analysed during the current study are available from the corresponding author on request.
