# Author Correction: Electromyographic activity of the vastus medialis and gastrocnemius implicates a slow stretch-shortening cycle during rowing in the field

**DOI:** 10.1038/s41598-020-73251-5

**Published:** 2020-09-23

**Authors:** Steffen Held, Tobias Siebert, Lars Donath

**Affiliations:** 1 Department of Intervention Research in Exercise Training, German Sport University Cologne, Cologne, Germany; 2 Department of Sport and Motion Science, University of Stuttgart, Stuttgart, Germany

Correction to: *Scientific Reports*, 10.1038/s41598-020-66124-4, published online 11 June 2020.

The original version of this Article contained errors in the spelling of the authors Steffen Held, Tobias Siebert & Lars Donath which were incorrectly given as Held Steffen, Siebert Tobias & Donath Lars respectively.

These errors have now been corrected in the PDF and HTML versions of the Article.

